# Prevalence of exposure to benzodiazepines among pregnant women in Taiwan: A nationwide longitudinal study

**DOI:** 10.1111/jsr.13678

**Published:** 2022-07-01

**Authors:** Yu‐Hsuan Lin, Mei‐Huei Chen, Ya‐Chen Chang, Likwang Chen, Chao A. Hsiung, Shiow‐Ing Wu

**Affiliations:** ^1^ Institute of Population Health Sciences National Health Research Institutes Miaoli County Taiwan; ^2^ Department of Psychiatry National Taiwan University Hospital Taipei Taiwan; ^3^ Department of Psychiatry, College of Medicine National Taiwan University Taipei Taiwan; ^4^ Institute of Health Behaviors and Community Sciences, College of Public Health National Taiwan University Taipei Taiwan; ^5^ Department of Pediatrics National Taiwan University College of Medicine and Hospital Taipei Taiwan

**Keywords:** benzodiazepine, benzodiazepine‐like Z‐hypnotics, defined daily dose, pregnancy, prevalence

## Abstract

Although more than one hundred studies have examined the prevalence of the use of benzodiazepines and benzodiazepine‐like Z‐hypnotics (BZDs) among pregnancy events, further analysis of the effects of dosage or type of BZDs is needed. The aim of this study was to examine the prevalence rate of BZDs use in pregnancy events, stratified by trimester over time, with characteristics of the dosage and type of BZDs. This is a retrospective population study based on linking three national databases. We examined the prevalence rates from 2004 to 2017, and contrasted the results based on >0 defined daily dose (DDD) and ≥0.5 DDD. We identified 2,630,944 pregnancy events with live births; 89,897 (3.4%) of the associated pregnancy events had used some form of BZD during pregnancy. The prevalence of BZDs use, as defined by >0 DDD, decreased from 4.1% in 2004 to 2.9% in 2017, indicating a decrease in sporadic use and an increase in stable use within therapeutic doses. Meanwhile, BZDs use defined by ≥0.5 DDD increased from 0.1% in 2004 to 0.4% in 2017. Zolpidem was the most frequently prescribed BZDs, as defined by >0 DDD or ≥0.5 DDD. This national cohort study demonstrates the importance of average dosage in the definition of BZDs use in pregnancy events, and it found opposite trends in the prevalence of use between different dosages.

## 1 INTRODUCTION

Benzodiazepines and benzodiazepine‐like Z‐hypnotics (BZDs) are generally prescribed for the treatment of sleep disturbances and anxiety disorders (Brunton, Hilal‐Dandan, & Knollmann, 2018; Shyken, Babbar, Babbar, & Forinash, 2019). These medications have anxiolytic, hypnotic, muscle relaxant, and anticonvulsant properties, and may relieve symptoms in the short‐term (Soyka, 2017). When used during pregnancy, these medications cross the placental and blood–brain barrier, where they can bind to γ‐amino butyric acid receptors in the developing fetal central nervous system, potentially affecting fetal growth and development (Briggs, Freeman, & Yaffe, 2012; Guerre‐Millo et al., 1979; Mandelli et al., 1975). Therefore, these drugs have been categorised according to their risk during pregnancy and lactation (Howland, 2009). Most BZDs, such as lorazepam, oxazepam, and diazepam, are categorized as D by the U.S. Food and Drug Administration, as well as by the Taiwan Food and Drug Administration (Food and Drug Administration, 2021), indicating that there is evidence of human fetal risk (Okun, Ebert, & Saini, 2015); Z‐hypnotics, such as zolpidem and zopiclone, are categorised as C, indicating that use is warranted; and estazolam is categorised as X, indicating that use is contraindicated.

Anxiety disorders and depression occur in up to 15% (Dennis, Falah‐Hassani, & Shiri, 2017) and 10% of pregnancy events, respectively (Vigod, Wilson, & Howard, 2016). In Taiwan, pregnant women were reported to experience both objective and subjective sleep disturbances during the early trimester (Tsai, Lee, Gordon, Cayanan, & Lee, 2021), with a substantial proportion of them also having high depressive symptoms throughout the pregnancy (Tsai, Lin, Wu, Lee, & Lee, 2016). While treatment recommendations and prescribing patterns for those conditions have shifted away from BZD therapy to antidepressants in recent years (Berney, Halperin, Tango, Daeniker‐Dayer, & Schulz, 2008; Offidani, Guidi, Tomba, & Fava, 2013), BZDs continue to be used frequently for pregnant women with recalcitrant symptoms associated with mental illness and pain, such as anxiety and insomnia. A recent meta‐analysis showed that 1.9% of pregnant women used BZDs (Bais et al., 2020). In Taiwan, the one‐year prevalence of BZDs use was 20% of adults (Wang, Chen, Chen, Chou, & Chou, 2014), which was higher than in other countries, such as the prevalence of 5.2% in United States (Olfson, King, & Schoenbaum, 2015). However, the prevalence of pregnant women using BZDs by a specific dosing was unknown.

A systematic review of more than 178 studies on the prevalence of BZDs usage in pregnant women found no information on dosing of prescriptions (Bais et al., 2020). The precise BZDs dosing prescribed to pregnant women is unknown. The systematic review showed no cohorts that reported prevalence rates over a series of subsequent calendar years. In addition, to date, clear drug‐specific data on the use of BZDs during pregnancy remains unknown. We used data from the national population‐based cohort study linked with data from NHI database, Maternal and Child Health database, and the Birth Certificate Application to examine (1) the prevalence rate of BZDs over time; (2) prescription behaviours, such as dosing and the types of these medications, and during which trimester these medications were received; and (3) characteristics of patients, such as history of mental illness.

## 2 METHODS

### 2.1 Study setting and ethical approval

This is a retrospective population study based on data from Taiwan's Health and Welfare Data Science Centre (HWDC; https://dep.mohw.gov.tw/DOS/lp-2503-113-xCat-DOS_dc004.html), a large data centre built by Taiwan's Ministry of Health and Welfare. The study protocol was approved by the Research Ethics Committee of the National Health Research Institutes (EC1050602). Because no identifiable personal data could be accessible, the need for informed consent was waived.

All BZDs are class III or class IV controlled drugs in Taiwan (Food and Drug Administration, 2022). Most of them were categorised as D by the Taiwan Food and Drug Administration, indicating that there is evidence of human fetal risk. All medical doctors could prescribe BZDs for pregnant women, and the Taiwanese physicians typically followed the warnings on the package inserts, such as to avoid using BZDs for pregnant women, and the duration should be no longer than 2 to 4 weeks. The prescribing restriction for non‐psychiatry specialists included that the dosage be no more than one tablet per day, and the duration was limited to no more than 6 months. If the patients were indicated to long‐term use of BZDs, the medical doctors have to record the reason and refer to psychiatrists when necessary. For patients who had not established a stable therapeutic alliance, the prescription for BZDs was limited to no more than 7 days (National Health Insurance Administration, 2022).

### 2.2 Data sources

The major database for this study is Taiwan's National Health Insurance (NHI) database. Taiwan started its National Health Insurance Programme in 1995 and has reached 99% coverage since 2004 (Lin, Gash, Smeeth, & Chen, 2018). This is a rich source of data for epidemiological and clinical research (Hsieh et al., 2019; Huang et al., 2021; Liao, Ko, Chen, & Hsiao, 2020; Sung, Hsieh, & Hu, 2020). The NHI system codes diagnoses using the International Classification of Diseases, Ninth Revision (ICD‐9) or Tenth Revision (ICD‐10). The database we acquired contained individual‐level longitudinal NHI claims and registration data for the entire national population for the years from 2004 to 2017. To construct an integrated database for pregnancy events who had live births in these 14 years, we linked three national databases: the NHI database, the Maternal and Child Health database, and the Birth Certificate Application database.

### 2.3 Study participants and **BZDs** dosing measurement

**FIGURE 1:**
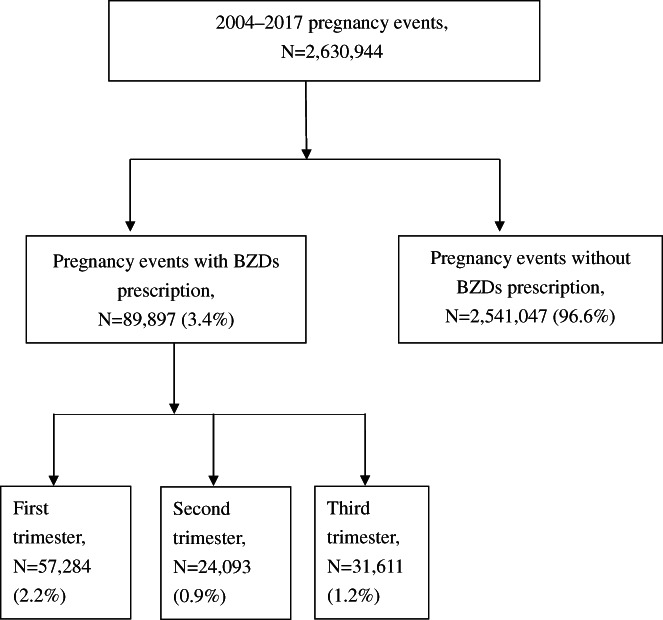
Flowchart of study participant selection

We identified 2,630,944 pregnancy events with live births (Figure 1). On the basis of birth certificate data, we identified a date of conception for each delivery event by referring to gestational age. Subsequently, we identified three trimesters for each birth event: the first (conception until day 90), second (the subsequent 90 days), and third (day 181 until delivery). Because each birth certificate provides information on gestational age, we could identify full‐term and non‐full‐term babies. For pregnancy events with different gestational periods, the third trimesters (day 181 until delivery) had different numbers of days. We used this method for pregnancy events in all study years. In this study, each observational record reflected a pregnancy event. Thus, for a woman who had live births from multiple pregnancy events in years from 2004 to 2017, the database included multiple case records for this woman. In contrast, for a pregnancy event with multiple live births, the database had only one case record.

On the basis of the Anatomical Therapeutic Chemical Classification System, we categorised BZDs into 26 groups for BZD dosing estimation and comparison purposes, excluding injection forms because those are usually prescribed for muscle‐relaxant or anticonvulsant properties rather than as anxiolytics or hypnotics. By referring to data on the dates that the prescriptions were issued, as well as to the amounts and the frequencies of medication use indicated on the prescriptions, we calculated the cumulative prescribed BZD dosing for each pregnancy event during each of the three trimesters and the whole pregnancy period. We then calculated the average daily BZD dosing for a specific period for a woman by dividing the cumulative BZD dosing by the number of days in that period. We estimated and expressed the BZD dosing according to the defined daily dose (DDD) system constructed by the World Health Organization (WHO Collaborating Centre for Drug Statistics Methodology, 2022). Although the DDD system only measures the total exposure of BZDs rather than the frequency, the DDD is a unit developed to measure the average maintenance dose per day for a drug used for its main indication in adults, especially in the registered database study. We used the cutoff value of 0.5 DDD/day during a period of pregnancy to classify BZDs users as sporadic users and regular users based on the widely used definition of 0.5 DDD/day for 180 days or a year as the “long‐term BZDs use” (Fang et al., 2009; Zandstra et al., 2002), or the “frequent BZDs” (Andenæs, Helseth, Misvær, Småstuen, & Ribu, 2016).

The list of BZDs items investigated in this study is as follows: zolpidem, flunitrazepam, alprazolam, zopiclone, lorazepam, estazolam, brotizolam, triazolam, flurazepam, fludiazepam, bromazepam, midazolam, nitrazepam, diazepam, zaleplon, oxazolam, clobazam, oxazepam, lormetazepam, nimetazepam, nordazepam, eszopiclone, chlordiazepoxide, clorazepate dipotassium, cloxazolam, and medazepam.

### 2.4 Medication use prevalence estimation and subgroup comparison

We further constructed the definition of a medication user in order to investigate the prevalence of BZD usage during pregnancy. We adopted two definitions for comparison purposes: one regarded women with any use (>0 DDD) as users, the other included only women with an average daily exposure level greater than 0.5 DDD as users. We examined the prevalence rates across years, and contrasted the results based on the two definitions regarding medication users.

We also conducted subgroup comparisons to explore whether a woman's history of mental illness would be associated with medication‐use intensity. We screened all NHI records of outpatient visits and the inpatient medical record for each pregnancy event in two periods: the 1 year immediately before conception, and the pregnancy period. For each period, a woman was regarded as one with a history of mental illness if she had a diagnosis of mental illness in no fewer than three outpatient visits or at least one hospitalisation. Disease codes for identifying mental illness are as follows: 290–319 in the system of ICD‐9‐CM, and F00‐F99 in ICD‐10‐CM. Subsequently, we categorised women into the following four groups to conduct further comparison: (1) no mental illness in either period, (2) mental illness in the year before conception but no illness during pregnancy, (3) no mental illness in the year before conception but some illness during pregnancy, and (4) mental illness in both periods.

### 2.5 Statistical analysis

We used SAS software version 9.4 (SAS Institute Inc., Cary, NC) to extract and organise study participants' data, and conducted statistical analysis subsequently. Research variables were reported as mean ± standard deviation (SD) for continuous variables and as percentages for categorical variables. To investigate how often these BZDs items were prescribed, we ranked BZDs items by comparing the proportions of pregnancy events fulfilling the definition of BZDs use for various BZDs items among all pregnancy events examined in the study. We used linear regression to examine the trends in the prevalence of medication use. For subgroup comparison, we adopted the chi‐square test to examine the proportions of BZDs users.

## 3 RESULTS

In this study, the relevant data associated with 2,630,944 cases of pregnancy events were recruited according to the above criteria. The mean and standard deviation (SD) of age at pregnancy were 30.5 ± 4.9 years. Among the pregnancy events, 89,897 (3.4%) had taken any BZDs during pregnancy. Their average BZD intake was 0.2 (SD = 0.7) DDD. The distribution in the first, second, and third trimesters were 57,284 (2.2%), 24,093 (0.9%), and 31,611 (1.2%), respectively (Figure 1). Among these BZDs prescriptions, 20.9% were prescribed by psychiatrists and the other 79.1% were prescribed by other medical specialties.

**FIGURE 2:**
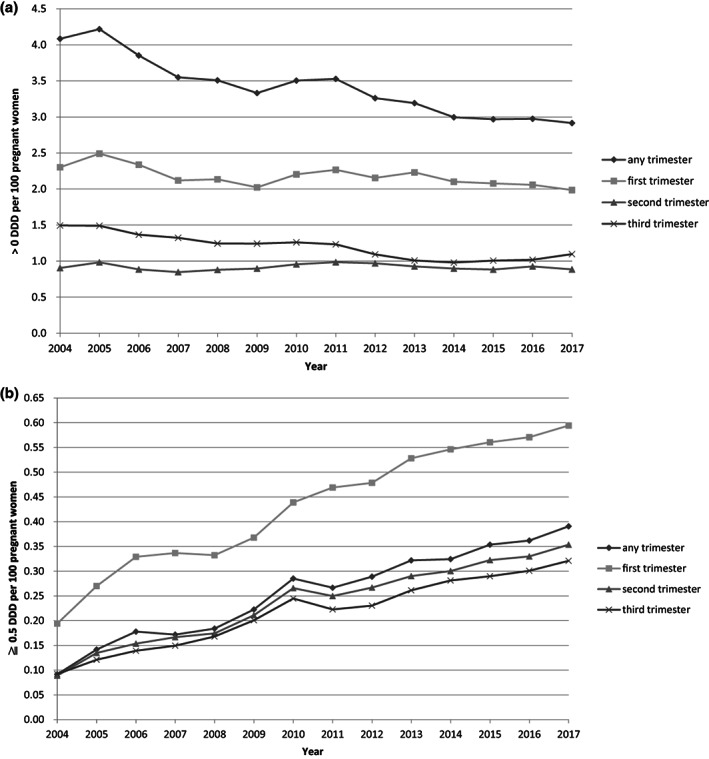
(a) Prevalence of benzodiazepines among >0 DDD, stratified by pregnancy trimesters in Taiwan, 2004–2017 linear regression was used to examine the trends in the prevalence of medication use. *R* ^2^ for (1) any trimester: 0.8894, (2) first trimester: 0.479, (3) second trimester: 0.0002, (4) third trimester: 0.8575 *p* value for (1) any trimester: <0.0001, (2) first trimester: 0.006, (3) second trimester: 0.964, (4) third trimester: <0.001. (b) Prevalence of benzodiazepines among ≥0.5 DDD, stratified by pregnancy trimesters in Taiwan, 2004–2017 the linear regression was used to examine the trend in the prevalence of medication use. *R* ^2^ for (1) any trimester: 0.9695, (2) first trimester: 0.9653, (3) second trimester: 0.971, (4) third trimester: 0.9702 *p* value for (1) any trimester: <0.0001, (2) first trimester: <0.001, (3) second trimester: <0.001, (4) third trimester: <0.001

Figure 2a shows the prevalence of any BZD usage during pregnancy. For pregnancy events with the use of BZDs >0 DDD in any trimester, the prevalence decreased from 4.1% in 2004 to 2.9% in 2017. (The downward trend in the prevalence rate was 0.09% per year, *R* ^2^ = 0.8894, *p*‐value <0.0001). When stratified by three trimesters, the highest prevalence of any BZDs usage occurred in the first trimester, followed by the third and second trimesters. The corresponding prevalences during the study period were between 2.0% to 2.5%, 1.0% to 1.5%, and 0.8% to 1.0%, respectively. The prevalence of BZDs >0 DDD in first trimester was 2.3% in 2004 and 2.0% in 2017, respectively.

Among pregnancy events with the use of BZDs ≥0.5 DDD, the prevalence of BZDs use during pregnancy showed an upward trend during the study period (Figure 2b). In any trimester, the prevalence increased from 0.09% in 2004 to 0.4% in 2017; the upward trend in the prevalence rate was 0.02% per year, *R* ^2^ = 0.9695, *p*‐value <0.0001. The patterns of increase in prevalence were similar across trimesters. The prevalences of BZDs usage ≥0.5 DDD in the first, second, and third trimesters of 2004 were 0.2%, 0.09%, and 0.09%, respectively, while the corresponding prevalences in 2017 were 0.6%, 0.4%, and 0.3%, respectively.

**FIGURE 3:**
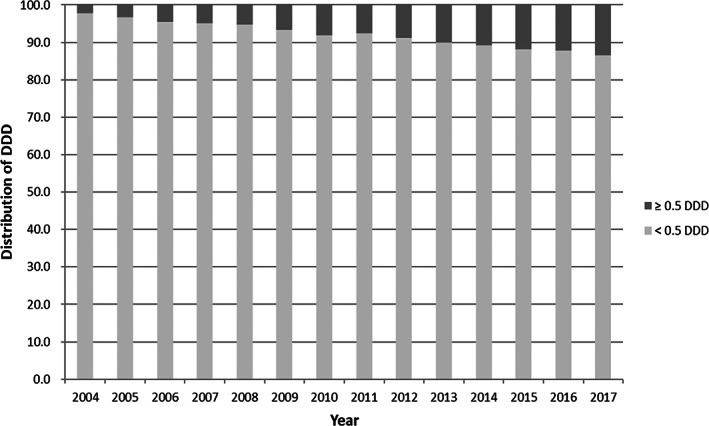
Proportion of benzodiazepine prescriptions by DDD during pregnancy in Taiwan, 2004–2017

Figure 3 shows the proportion of BZDs prescriptions by DDD during pregnancy. The majority of BZDs prescriptions were less than 0.5 DDD, with the figure dropping from 97.8% in 2004 to 86.6% in 2017. However, the proportion of BZDs use with ≥0.5 DDD increased from 2.2% in 2004 to 13.4% in 2017, respectively.

Table 1 shows the ranking of BZDs prescription during pregnancy with stratification by DDD. Regardless of the dosage, zolpidem ranked first in use of BZDs during pregnancy. The top seven BZDs with dosage ≥0.5 DDD were zolpidem (prescription proportion = 59.1%), flunitrazepam (16.3%), alprazolam (10.0%), zopiclone (4.3%), lorazepam (3.7%), estazolam (3.1%), and brotizolam (1.5%). Five of those — zolpidem, alprazolam, lorazepam, flunitrazepam, and estazolam — were also among the top seven BZDs with dosage >0 DDD. Diazepam ranked 3rd in stratification with BZDs dosage >0 DDD but dropped to the 14th position in the stratification with BZDs dosage ≥0.5 DDD. Although estazolam is listed as a drug contraindicated during pregnancy, it still ranked 6th in the stratification with BZDs dosage ≥0.5 DDD.

**TABLE 1:** Ranking of benzodiazepine prescription stratified by DDD during pregnancy

Ranking	Proportion (%)	>0 DDD	Ranking	Proportion (%)	≥0.5 DDD
1	36.2	Zolpidem	1	59.1	Zolpidem
2	16.8	Alprazolam	2	16.3	Flunitrazepam
3	16.1	Diazepam	3	10.0	Alprazolam
4	15.5	Lorazepam	4	4.3	Zopiclone
5	9.3	Oxazolam	5	3.7	Lorazepam
6	8.4	Fludiazepam	6	3.1	Estazolam
7	4.5	Estazolam	7	1.5	Brotizolam
8	3.9	Bromazepam	8	1.2	Triazolam
9	2.8	Flunitrazepam	9	1.0	Flurazepam
10	2.4	Zopiclone	10	0.6	Fludiazepam
11	2.3	Medazepam	11	0.5	Bromazepam
12	2.0	Nitrazepam	12	0.4	Midazolam
13	1.1	Chlordiazepoxide	13	0.4	Nitrazepam
14	1.0	Midazolam	14	0.4	Diazepam
15	0.9	Triazolam	15	0.4	Zaleplon
16	0.8	Brotizolam	16	0.3	Oxazolam
17	0.6	Flurazepam	17	0.3	Clobazam
18	0.5	Oxazepam	18	0.2	Oxazepam
19	0.4	Zaleplon	19	0.1	Lormetazepam
20	0.3	Nordazepam	19	0.1	Nimetazepam
21	0.2	Clobazam	21	0.1	Nordazepam
22	0.2	Lormetazepam	22	0.06	Eszopiclone
23	0.06	Nimetazepam	23	0.01	Chlordiazepoxide
24	0.05	Clorazepate dipotassium	24	0.01	Clorazepate dipotassium
25	0.02	Eszopiclone	25	0.0	Cloxazolam
26	0.003	Cloxazolam	25	0.0	Medazepam

Proportion (%) is calculated by dividing the numbers of pregnancy events with each specific BZD prescription by the numbers of pregnancy events with the BZD prescription during pregnancy from 2004 to 2017.

There were significant differences in the association between mental illness diagnosed before and during pregnancy and DDD prescription of BZDs during pregnancy (Table 2). In the study population, 2,574,173 pregnancy events had no diagnosis of mental illness either before or during their pregnancy. Among them, 97.3% did not receive any BZDs prescription during pregnancy. For women who were diagnosed with mental illness before and during pregnancy, only about 38.8% (3739/9626) took BZDs ≥0.5 DDD during pregnancy. Of the 6730 pregnancy events with use of BZDs ≥0.5 DDD during pregnancy, 4413 (65.6%) were diagnosed with mental illness during pregnancy.

**TABLE 2:** The association between mental illness diagnosed before and during pregnancy and benzodiazepine prescription during pregnancy (with stratification by DDD)

Mental illness		During pregnancy							
1 year before pregnancy	During pregnancy	No use		>0 DDD – <0.5 DDD		≥0.5 DDD		Total	
		No.	%	No.	%	No.	%	No.	*p* Value
No	No	2,503,431	97.3	69,058	2.7	1684	0.1	2,574,173	<0.0001
Yes	No	31,541	77.9	8294	20.5	633	1.6	40,468	
No	Yes	3700	55.4	2303	34.5	674	10.1	6677	
Yes	Yes	2375	24.7	3512	36.5	3739	38.8	9626	

*p*‐Values were assessed with chi‐square test.

## 4 DISCUSSION

### 4.1 Main findings

The present study leveraged the national cohort to examine 2,630,944 pregnancy events during a 14‐year period and found a decreased prevalence of BZDs usage during pregnancy, from 4.1% in 2004 to 2.9% in 2017. This is also the first study to define the prevalence and trends by different dosage of BZDs. We used the dosage ≥0.5 DDD to identify whether BZDs were dispensed for regular or as‐needed/sporadic use. The prevalence of 0.1% to 0.4% with a therapeutic dosage ≥0.5 DDD was lower than the global prevalence of 1.9% (1.6%–2.2%) or that of 0.9% (0.4%–1.5%) in Asian countries reported by a recent meta‐analysis (Bais et al., 2020). Zolpidem, a relatively safe hypnotic (category C), was the most frequently used/prescribed BZDs by the definition of >0 DDD or ≥0.5 DDD. A total of 0.1% women who did not have a history of mental illness were prescribed BZDs ≥0.5 DDD on average, while 38.8% of women with a diagnosis of mental illness both before and during pregnancy were prescribed BZDs ≥0.5 DDD on average.

### 4.2 Opposite trends of prevalence from different dosages of **BZDs**

The 29.3% decrease in BZDs use from 4.1% in 2004 to 2.9% in 2017, as defined by >0 DDD, consisted of both a decrease in sporadic use and an increase in stable use within therapeutic doses. The proportion of BZDs dosage <0.5 DDD, which represented sporadic use, decreased significantly, from 97.8% in 2004 to 86.6% in 2017. In addition, diazepam, which was prescribed mainly as a muscle relaxant and anticonvulsant for sporadic use rather than as a hypnotic or anxiolytic, was the 3rd most frequently used BZDs by the definition of >0 DDD, whereas it ranked 14th by the definition of ≥0.5 DDD. By contrast, the 300% increase in BZD use (from 0.1% in 2004 to 0.4% 2017), under the definition of ≥0.5 DDD, was consistent with the results of the only two previous cohort studies (Askaa, Jimenez‐Solem, Enghusen Poulsen, & Traerup Andersen, 2014; Martin, Mak, Miller, Welsh, & Terplan, 2015). This increase in the use of BZDs within a clinical therapeutic dose (usually ≥0.5 DDD) also paralleled an increase in diagnoses of common mental disorders (i.e., depression and anxiety) in Taiwan during the past 20 years (Fu, Lee, Gunnell, Lee, & Cheng, 2013).

Although our findings showed that the BZDs were more likely prescribed to women who were diagnosed with mental illness before and during pregnancy, the characteristics of claim data limited the validity to assess the indication of the prescribed BZDs in the present study. For example, insomnia is one of the common symptoms of generalised anxiety disorder and major depressive disorder, and sleep disorder is also a comorbid psychiatric disorder in postpartum mood disorder. A future study is needed to analyse the time varied individual‐level confounders such as the age of pregnancy, the diagnosis of the mental illness, and the detailed indication of the BZDs use.

The rank defined by dosage ≥0.5 DDD differed from the rank of dosage >0 DDD, suggesting a relatively stable prescribing behaviour of therapeutic doses of hypnotics or anxiolytics. The opposite trends by different definition of dosage in our study demonstrate the substantial heterogeneity in women who had been prescribed BZDs; the heterogeneous dosage might explain the findings in a previous meta‐regression, which did not show a significant change in BZD use over time among 7,343,571 pregnancies (Bais et al., 2020). We believe future studies of BZDs should include the dosage to estimate associated prevalences and trends in their use.

### 4.3 Clinical implications

Our cohort showed that during the 14‐year study period most prenatal BZDs use occurred in the first trimester, and the prevalence was highest in the first trimester by different definitions of DDD (>0 DDD: 2.2%, ≥0.5 DDD: 0.4%,). These results were similar to those of a recent large cohort study, which reported a rate of prenatal BZDs use of 2.5%, with 84% of use occurring in the first trimester (Yonkers, Gilstad‐Hayden, Forray, & Lipkind, 2017). Physicians should note carefully the history of the menstrual cycle and the possibility of pregnancy before prescribing BZDs to reproductive‐aged women. Clinicians should pay more attention to the use of BZDs in reproductive‐aged women. Of particular concern is that women may have exposure without awareness during the first trimester, when organogenesis is taking place. Of note, the literature is not consistent as to in which trimester the exposure would be more harmful for the fetus. On one hand, it is advised that drug use should be avoided during the first trimester, due to potential teratogenic risks (Iqbal, Sobhan, & Ryals, 2002), although these risks have thus far not been demonstrated by a meta‐analysis (Enato, Moretti, & Koren, 2011). On the other hand, it has also been mentioned that late‐third‐trimester use is associated with more risks for the fetus or neonate (McElhatton, 1994), including the risk of floppy infant syndrome, which could lead to hypoxia and even irreversible damage in the neonate (Dammann & O'Shea, 2008).

There are several implications of the ranking of the most frequently used BZDs with dosage ≥0.5 DDD. First, physicians should carefully inform patients in detail of the risks and benefits of BZD use during pregnancy, especially because most BZDs are categorised as having shown evidence of human fetal risk; of particular concern is estazolam, which is contraindicated in pregnant women, and yet was still the 6th most frequently used BZD in our national cohort. Second, although zolpidem was the most frequently used hypnotic during pregnancy, it is unknown whether Taiwanese physicians were aware that for pregnant women zolpidem is a relatively safe (category C) hypnotic. However, the Z‐drugs in category C, such as zolpidem and zopiclone, were still recommended for pregnant women rather than the most BZDs in category D. Third, since the top three most frequently used prenatal hypnotics — zolpidem, flunitrazepam, and alprazolam — were also the most frequently used hypnotics in general (Su et al., 2002), these prescribing patterns could reflect suboptimal risk–benefit evaluation in pharmacotherapies for pregnant women. In addition, Taiwan National Health Insurance data showed that 79.1% of BZDs were prescribed by non‐psychiatrist physicians, which was consistent with the patterns in the previous survey (Su et al., 2002). Although we showed that pregnancy events with a diagnosis of mental illness were more likely to receive hypnotics, our findings raise the question as to whether these practice patterns represent problems such as poor access to first‐line evidence‐based care, for instance, cognitive behaviour therapy for depression or anxiety or for insomnia. As well as the changing treatment guidelines in the treatment of depression and anxiety disorders with antidepressants instead of benzodiazepines (Berney et al., 2008; Offidani et al., 2013), where BZDs are used on an “as needed” basis.

### 4.4 Limitations

Several methodological limitations should be noted when interpreting our findings. First, studies using prescription records as a proxy for BZDs use may overestimate the actual use due to non‐compliance. Second, pregnancy was defined by only live‐born infants; but given that prenatal BZDs may be associated with loss (i.e., miscarriages and abortions), the prevalence of BZD use may have been underestimated. Third, different patterns of BZD use exist among the same average DDD, with some women using these BZDs on occasion and others chronically; as a result, different patterns of use may have diverse implications for both mother and fetus.

In conclusion, this national cohort study demonstrated the important role of average dosage in the definition of BZD use in pregnancy events, and we found opposing trends of prevalence between the different dosage definitions. The finding that the most frequent use was in the first trimester could, along with the uncovering of the rankings of specific BZDs, help to estimate exposure and to prioritise and guide future investigations.

## AUTHOR CONTRIBUTIONS

Conceptualization: Yu‐Hsuan Lin, Mei‐Huei Chen, Shiow‐Ing Wu.

Methodology: Yu‐Hsuan Lin, Mei‐Huei Chen, Li‐kwang Chen, Shiow‐Ing Wu.

Writing‐Original draft preparation: Yu‐Hsuan Lin, Mei‐Huei Chen, Li‐kwang Chen.

Formal analysis and investigation: Ya‐Chen Chang.

Writing‐Reviewing and Editing: Yu‐Hsuan Lin, Mei‐Huei Chen, Li‐kwang Chen, Shiow‐Ing Wu.

Supervision: Chao A. Hsiung, Shiow‐Ing Wu.

## FUNDING

This research was funded by the National Health Research Institutes, Taiwan (PH‐110‐PP‐22).

## CONFLICT OF INTEREST

This research was supported by a grant from the National Health Research Institutes (no. PH‐110‐PP‐22). The authors declare no potential conflicts of interest.

## Data Availability

The data for this study are not publicly available. To protect privacy, the Taiwanese government only allows researchers to analyze data from the Health and Welfare Data Science Center in selected computer rooms of the center. No individual data can be brought outside the center, and researchers can only bring away aggregate statistical results based on raw data. To use the data, a researcher has to apply for permission of using data within the center. To apply for such permission, a researcher has to submit an IRB approval concerning the data use, and has to pay for using data. The amount of payment depends on the volume of data used.
